# Neonicotinoids thiamethoxam and clothianidin adversely affect the colonisation of invertebrate populations in aquatic microcosms

**DOI:** 10.1007/s11356-017-1125-5

**Published:** 2018-01-22

**Authors:** Kate Basley, Dave Goulson

**Affiliations:** School of Life Sciences, The University of Sussex, Falmer, East Sussex, BN1 9QG UK

**Keywords:** Aquatic invertebrates, Neonicotinoids, Pesticides, Freshwater contamination

## Abstract

Surface waters are sometimes contaminated with neonicotinoids: a widespread, persistent, systemic class of insecticide with leaching potential. Previous ecotoxicological investigations of this chemical class in aquatic ecosystems have largely focused on the impacts of the neonicotinoid imidacloprid; few empirical, manipulative studies have investigated the effect on invertebrate abundances of two other neonicotinoids which are now more widely used: clothianidin and thiamethoxam. In this study, we employ a simple microcosm semi-field design, incorporating a one-off contamination event, to investigate the effect of these pesticides at field-realistic levels (ranging from 0 to 15 ppb) on invertebrate colonisation and survival in small ephemeral ponds. In line with previous research on neonicotinoid impacts on aquatic invertebrates, significant negative effects of both neonicotinoids were found. There were clear differences between the two chemicals, with thiamethoxam generally producing stronger negative effects than clothianidin. Populations of Chironomids (Diptera) and Ostracoda were negatively affected by both chemicals, while Culicidae appeared to be unaffected by clothianidin at the doses used. Our data demonstrate that field-realistic concentrations of neonicotinoids are likely to reduce populations of invertebrates found in ephemeral ponds, which may have knock on effects up the food chain. We highlight the importance of developing pesticide monitoring schemes for European surface waters.

## Introduction

The majority of species in freshwater aquatic ecosystems are arthropods. These are an essential link in the transfer of energy up the freshwater food chain, being a primary food source for many species of vertebrates, such as fish, amphibians and birds (Chagnon et al. 2015). A decrease in arthropod abundance or diversity is therefore likely to result in a loss of important ecosystem processes and knock-on effects for higher trophic levels (Covich et al. 2004; Hallmann et al. 2014).

Small-scale aquatic habitats such as temporary ponds and puddles often fulfil an important ecological role at the landscape level (De Meester et al. 2005). Similarly, ditches are crucial features for land drainage and, if managed properly, can also provide habitats for wildlife. Although such ephemeral habitats are the least species rich of the freshwater features in an agricultural landscape, they have been found to support a diversity of specialist temporary water invertebrates (Williams 2004). Nicolet et al. (2004), found that, of 71 temporary ponds surveyed in England and Wales, 75% of these supported at least one nationally scarce macro-invertebrate and 8% supported at least one nationally scarce plant species across a range of physico-chemical characteristics.

Globally, neonicotinoids have become the most widely used insecticides due in part to their systemic properties in the crop to be protected and also their relatively low-vertebrate toxicity (Jeschke et al. 2011). However, with the exception of the Netherlands, most countries in Europe and other parts of the world do not have a system in place for the systematic monitoring of neonicotinoid pesticides in aquatic systems, although the monitoring of pesticide presence in water is required under the European Drinking Water Directive (Allan et al. 2006). It has been shown that at the global scale, more than 50% of detected insecticide concentrations exceed regulatory levels, indicating that surface waters and therefore aquatic biodiversity are at risk of harm from current insecticide use (Stehle and Schulz 2015). In the UK, a 2-m protection zone must be left around ditches and watercourses in all fields of 2 ha or more to minimise water contamination (DEFRA 2006). However, the risk of contamination via neonicotinoid seed dressings is not currently addressed; the only stipulation in their use is that treated seeds are kept away from surface water, which does not account for the possibility of lateral movement of neonicotinoids through the soil profile nor movement of the pesticide in surface runoff.

There are widespread concerns as to their potentially far-reaching impacts upon wildlife (Chagnon et al. 2015; Goulson 2013; Hallmann et al. 2014; Pisa et al. 2015; Van Dijk et al. 2013; Whitehorn et al. 2012). Neonicotinoids and their toxic metabolites have been found to be persistent, not just in the target plant, but also in water, aquatic sediments and soil (van der Sluijs et al. 2013). A recent review concluded that low levels of neonicotinoids cause negative effects on aquatic ecosystems both at the individual and population level (Pisa et al. 2015), and the effect has been found to extend to zooplankton, benthic and neuston communities (Hayasaka et al. 2012).

The persistence of neonicotinoids increases the duration over which non-target organisms may be exposed (Krupke et al. 2012; van der Sluijs et al. 2013). Where the neonicotinoid is used as a seed dressing, studies have shown that only 1.6–20% of the active ingredient is absorbed by the crop. The remainder is either lost as dust during sowing (approximately 1–2%) or enters the soil (typically more than 90%) (Tapparo et al. 2012). Due to their high runoff and capacity to leach into surface and ground waters (González-Pradas et al. 2002), neonicotinoids have often been detected in aquatic environments, including streams, lakes and temporary bodies of water such as puddles (Chagnon et al. 2015).

Imidacloprid, one of the earlier most widely used neonicotinoids, has been found in the Netherlands in groundwater, streams and ditches at concentrations far exceeding the maximum allowable risk level (13 ng/l) and has also been detected in 89% of rivers, creeks and drains in California, 19% of those samples exceeding the US Environmental Protection Agency (EPA) guideline concentration of 1.05 ppb (Starner and Goh 2012). However, it is common for residue levels of neonicotinoids to be much lower; a survey of surface water contamination studies found clothianidin to be generally in the region of 0.003–3.1 ppb and thiamethoxam to be around 0.001–225 ppb (Morrissey et al. 2015). Surface waters, including puddles, ditches, irrigation channels and streams in or near farmland, have been found to be contaminated by neonicotinoids (Morrissey et al. 2015; Van Dijk et al. 2013; Samson-Robert et al. 2014, Main et al. 2014, Schaafsma et al. 2015). Contamination levels of various types of surface waters differ. For example, samples taken from within and around the perimeter of corn fields in Southwest Ontario detected residues of clothianidin (mean = 2.28 ppb, maximum = 43.60 ppb) and thiamethoxam (mean = 1.12 ppb, maximum = 16.50 ppb) in 100 and 98.7% of samples tested, respectively (Morrissey et al. 2015; Schaafsma et al. 2015). Streams near to fields of corn and soybean production contained median levels of 8.2 ppb of clothianidin and levels of < 2 ppb thiamethoxam (Hladik et al. 2014). Both thiamethoxam and clothianidin have relatively long half-lives in soil; the DT50 of clothianidin is 148–1155 days, and thiamethoxam’s is 229 days on average (Main et al. 2014). Their persistence in the soil and high-water solubility (thiamethoxam = 4100 mg/L; clothianidin = 327 mg/L (Main et al. 2014)) means there is high potential to be transported into surface waters.

A significant negative relationship between imidacloprid polluted surface water and macro-invertebrate abundance has been found, after accounting for land-use differences between sites (Van Dijk et al. 2013). The authors found that macro-fauna abundance dropped off sharply between 0.013 and 0.067 ppb imidacloprid, concentrations more than an order of magnitude below the EPA guidelines. The results of an extensive review of laboratory and semi-field microcosm studies indicate that aquatic invertebrates are highly sensitive to neonicotinoids (Pisa et al. 2015). However, most of the studies were conducted using imidacloprid, a compound that is now relatively little-used (Goulson 2013), having been largely replaced by clothianidin or thiamethoxam (Defra 2014). There is thus a need to further investigate the impacts of these newer neonicotinoids on aquatic ecosystems. Here, we experimentally test the effect of field-realistic doses of clothianidin and thiamethoxam on the colonisation and development of aquatic invertebrate populations in puddle-replicate microcosms in semi-field conditions.

## Method

### Microcosm setup

Temporary water bodies were simulated by filling 14 L plastic buckets with 400 g of loamy soil and 10 L of either untreated or treated water (henceforth described as “microcosms”). The relative simplicity of the microcosm design allows temporary aquatic ecosystems to be created with high levels of replication (De Meester et al. 2005). Soil was collected from a single site, with no history of neonicotinoid usage, on the University of Sussex campus on the 20th August 2014. The soil was thoroughly mixed using a clean spade before being divided into 400 g samples which were placed in the clean buckets, these were left in the laboratory overnight.

In total, 140 microcosms were created on the 21st August 2014; 20 were controls, while ten microcosms were used for each of the following concentrations: 0.1, 1, 3, 7, 10 and 15 ppb of either thiamethoxam or clothianidin*.* Stock solutions were produced from analytical grade clothianidin and thiamethoxam (Sigma-Aldrich, Gillingham, UK) and made up in deionised water as they did not need to be stored. The concentrations of 0.1, 1 and 3 ppb were used to replicate levels that may be present in surface water due to chronic contamination after rain fall and leaching. The concentrations of 7, 10 and 15 ppb were used to replicate a singular pulse contamination, i.e. a rainfall event immediately after the sowing of a treated crop, before the active compound has bound to soil particles. Concentrations of clothianidin and thiamethoxam used in this experiment were within the ranges detected in a review of surface water samples (Morrissey et al. 2015). The buckets were filled with 10 L of fresh tap water and then dosed with neonicotinoid to create the contaminated microcosms. Once dosed, the soil and water fraction were thoroughly mixed. The microcosms were placed immediately adjacent to one another on a strip of grassland between two buildings in a 28 × 5 randomised block and were left uncovered to allow for colonisation by flying insects. This meant that the microcosms were subject to rainfall but this did not lead to overflow, and no one microcosm was subject to more rainfall than another. Microcosms were left in situ for 33–38 days.

### Data collection

As the microcosms had been filled to 10 L with fresh tap water and dosed straight after setup, it was expected that the population of aquatic invertebrates in the microcosms at the start of the experiment would be zero. The ostracoda subsequently detected in the microcosms were likely to have been presented as eggs in the soil, but were assumed to be evenly distributed as a result of the thorough mixing at the setup stage.

Commencing on 23rd September 2014, the invertebrate composition of the microcosms was quantified in a random order, over a 5-day period, using a random number generator*.* The water fraction was slowly poured through rinsed muslin in order to collect the live aquatic organisms that remained at the end of the experimental period, these were then stored in ethanol. The soil was rinsed through a 2-mm sieve to remove the larger stones and collected in a 250-μm sieve underneath in 100 g sub-samples to allow thorough searching for invertebrates. To collect the Chironomids, the sieve with the soil sample was slowly submerged so that Chironomid larvae floated to the surface; these were collected in a small hand-held sieve and stored in the ethanol.

The samples were subsequently drained through a 125-μm sieve to separate the organisms from the ethanol, which were then rinsed with deionised water. The sample was placed onto a white plastic tray marked with a grid; a small amount of water was added, and the tray was gently shaken to distribute the sample across the grid. The organisms present were identified and counted by eye. Identification was to subclass for aquatic mites (Acari), order for Ostracoda and family for Chironomidae and Culicidae. After counting, the sample was retained in ethanol for reference. Two control microcosms were lost due to sampling error.

### Statistical analysis

All statistical analyses were conducted using IBM SPSS Statistics 22. Too few Acari were detected for statistical analysis. The control replicates were pooled. Non-parametric Kruskal-Wallis H tests were preferred for this variable due to the significant heterogeneity found across the four population’s abundance data. These were used to test for significant differences across ranked means in the four populations (Chironomidae, *Culex* larvae, *Culex* pupae and Ostracoda) between groups of seven concentrations (control *n* = 18, 0.1, 1, 3, 7, 10 and 15 ppb all *n* = 10.) Post hoc Dunn’s tests with Bonferroni correction were used to determine significant differences between concentrations for each concentration group and for each neonicotinoid.

## Results

**Fig. 1:**
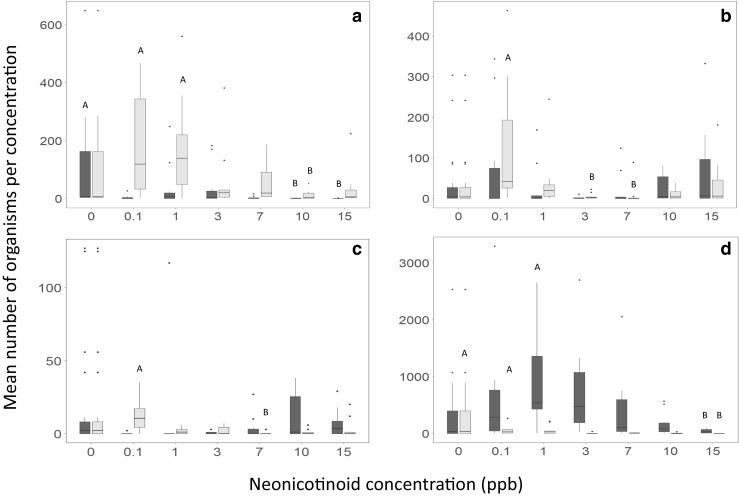
Effect of thiamethoxam (light grey) and clothianidin (dark grey) on mean population of aquatic invertebrates, for each separate neonicotinoid, means labelled A are significantly different than those labelled B; Dunn’s with Bonferroni correction. A-Chironomidae: thiamethoxam (*X*^2^ (6) = 16.1, *p* = 0.013)); 0.1 ppb − 10 adj. *p* = 0.036, 1–10 ppb adj. *p* = 0.048; clothianidin (*X*^2^ (6) = 21.9, *p* = 0.001)); control—10 ppb adj. *p*= 0.048, control—15 ppb adj. *p* = 0.003). B-Culex larvae: thiamethoxam (*X*^2^ (6) = 20.8, *p* = 0.002)); 0.1–3 ppb adj. *p* = 0.031, 0.1–7 ppb adj. *p* = 0.001; clothianidin—no statistically significant relationship existed between concentration and population abundance despite numbers dipping at 3 and 7 ppb (*p* = 0.498). C—Culex pupae: thiamethoxam (*X*^2^ (6) = 14.8, *p* = 0.021)); 0.1–7 ppb adj. *p* = 0.021; clothianidin (*X*^2^ (6) = 14.5, *p* = 0.025)); despite a statistically significant relationship overall, post hoc results showed no overall difference between means of each concentration replicate group when examining adjusted significance. D—Ostracoda: thiamethoxam (X^2^ (6) = 20.46, *p* = 0.002)); control—15 ppb adj. *p* = 0.033, 0.1–15 ppb adj. *p* = 0.029; clothianidin (*X*^2^ (6) = 17.6, *p* = 0.007)); 1–15 ppb adj. *p* = 0.023

Invertebrate populations in the microcosms contaminated with thiamethoxam showed significant differences across concentrations, with a general pattern of reduced numbers at higher concentrations of insecticide (Fig. 1), apart from *Culex* larvae whose numbers were highest at both the lowest and the highest concentrations. Ostracod numbers tended towards greater abundance in the low concentrations, with the greatest numbers being found in the control group; pairwise comparisons showed a significant difference between the control and 0.1 and 15 ppb (adj. *p* = 0.033 and 0.029, respectively).

Chironomidae, *Culex* pupae and Ostracoda showed a significant response to clothianidin concentration (Fig. 1), yet patterns for clothianidin were a little less clear than for thiamethoxam. For Chironomidae, the lowest abundance was found at the three highest clothianidin concentrations, with significant pairwise relationships between the control and the two highest concentrations (10 ppb adj. *p* = 0.048, 15 ppb adj. *p* = 0.003). Interestingly for clothianidin, low concentrations (0.1, 1 and 3 ppb) supported more Ostracod individuals than the controls, a pattern not replicated for thiamethoxam. A significant difference was noted between 1 and 15 ppb (adjusted *p* = 0.023). *Culex* larvae exhibited no statistically significant relationship between concentration and abundance.

## Discussion

Our data show that field-realistic concentrations of two commonly used neonicotinoids, thiamethoxam and clothianidin, significantly impact on populations of invertebrates (Diptera and Ostracoda) colonising aquatic microcosms, with some differences between the effects of the two chemicals. The aquatic microcosms were colonised mainly by flying Diptera (*Culex* and Chironomidae) which oviposited in the water, and also by Ostracoda, which may have originated from the soil added to each microcosm (they can survive for long periods in soil as desiccation-resistant eggs) (Özuluğ and Suludere 2012)). Organisms were found to differ in their sensitivity to both the concentration and particular class of neonicotinoid.

In a review of 214 toxicity tests including acute and chronic tests for neonicotinoids, Chironomidae were amongst the most sensitive taxa with many species exhibiting short-term lethal effects at clothianidin water concentrations of 1–29 μg/l (EC 2005 Summary; reviewed in Morrissey et al. 2015). A significant effect of thiamethoxam was observed on *Culex* pupae and *Culex* larvae; the relationship for *Culex* larvae was absent in the clothianidin microcosms which could be due to the higher concentrations of clothianidin delaying the development of the larvae, this effect has also been found in *C. riparious* exposed to thiamethoxam (Saraiva et al. 2017). Work by Sánchez-Bayo and Goka (2006) found that for the three freshwater Ostracod species investigated, 48 h LC_50_ was in the range of 301–715 μg/L for imidacloprid, far higher than the levels used here. However, the immobilisation bioassays for the same species were calculated to be in the range of 11–22 μg/L (24 h) and 5–7 μg/L (48 h), and clearly if such sub-lethal effects occurred in our microcosms then we would also expect impairment of feeding and reproduction due to the similar toxicity levels for aquatic organisms and identical mode of action of neonicotinoids (Morrissey et al. 2015).

It is possible that the actual final concentrations of neonicotinoids to which invertebrates were exposed in our microcosms were lower than those with which the water was originally dosed. Neonicotinoids are subject to rapid photolysis in clear water, and our microcosms were placed in a well-lit position in late summer. However, toxicity tests for imidacloprid performed under light or dark conditions have shown that LC50 values were not significantly different for any of the ostracod or cladoceran species tested; there is evidence to suggest that photolytic half-lives are difficult to relate to the actual persistence of neonicotinoids in natural waters (Sánchez-Bayo and Goka 2006), but our results should be interpreted with this caveat in mind. Of course degradation of pesticides following pulse contamination events would be expected in real water bodies in the field, so in this sense our microcosms are field-realistic.

The microcosms were also open to rainfall which would have diluted the pesticides, as it would concentrations in natural puddles. However, neonicotinoids persist for much longer in soils, so it is likely that they persisted in the soil fraction of the microcosm habitat. The difference in soil affinity of the two compounds could explain some of the observed differences in response between organisms (Morrissey et al. 2015). It is possible that after contamination with the pesticides, clothianidin bound to the soil fraction of the microcosm to a greater degree than thiamethoxam, and therefore less clothianidin was active in the water fraction. It is also possible that the more rapid photolysis of clothianidin (Morrissey et al. 2015) might have reduced its concentration in the water to a greater degree than that of thiamethoxam, potentially explaining the absence of a measurable effect on *Culex* larvae*,* which inhabit the open water. It is important to note that thiamethoxam degrades to clothianidin, so organisms are exposed to the toxic mixture for longer because the parent compound (thiamethoxam) is more stable in water, while the metabolite clothianidin is more persistent in soil (Morrissey et al. 2015); so the overall exposure is longer than if the organisms were only exposed to clothianidin.

It should be noted that our study does not attempt to distinguish between effects of the pesticides on colonisation of the microcosms and subsequent toxicological impacts on invertebrates. Reduced numbers of dipteran larvae could be due to either of these processes as Easton and Goulson (2013) report avoidance of pan-traps containing solutions of imidacloprid well below 1 ppb by dipterans. However, Ostracoda do not fly and it seems likely that they were in the soil placed into the buckets at the beginning of the experiment. The significant relationship between Ostracod number and increasing thiamethoxam and clothianidin concentration is therefore likely to be due to the toxicity of the compounds and not to any avoidance behaviour exhibited by this invertebrate.

Our data corroborate previous studies which suggest that neonicotinoids are likely to be broadly impacting aquatic invertebrates (Main et al. 2016; Mohr et al. 2012; Pestana et al. 2009a, b). All previous microcosm studies of this nature have studied the impacts of imidacloprid; we show that a single contamination at time-zero of a novel temporary water body by field-realistic levels of either thiamethoxam or clothianidin has a detrimental effect on the development of invertebrate populations, and invertebrates already present in the soil.

Van Dijk et al. (2013) describe broad patterns of reduced abundance of aquatic invertebrates in the Netherlands in permanent aquatic habitats where imidacloprid concentrations exceeded 13 ng/L. Such an effect has the potential to change the structure of the food web by affecting the population levels of the base organisms and therefore the transfer of energy to consumers (Chagnon et al. 2015). The knock-on and potential cascading effects of a neonicotinoid presence in freshwater have been indicated by Hallmann et al. (2014), who demonstrated that depletion of insect food resources caused by pollution of aquatic habitats had a negative impact on insectivorous passerine bird species in the Netherlands. Areas where imidacloprid concentrations in surface water were more than 20 ng/L saw the bird population decline by an average of 3.5% annually, for a period of 20 years. The invertebrates that inhabit temporary ponds are also an important food for vertebrate predators such as bats and birds, so our data add to the growing evidence that pollution of aquatic habitats may be contributing to cascading impacts on higher trophic levels**.** The data collected in this study further emphasises that there is a clear and pressing need for more extensive monitoring of pollution of aquatic habitats with neonicotinoids to allow us to properly evaluate the scale of this threat.
